# The Differential Organization of F-Actin Alters the Distribution of Organelles in Cultured When Compared to Native Chromaffin Cells

**DOI:** 10.3389/fncel.2017.00135

**Published:** 2017-05-04

**Authors:** Yolanda Gimenez-Molina, José Villanueva, Carmen Nanclares, Inmaculada Lopez-Font, Salvador Viniegra, Maria del Mar Francés, Luis Gandia, Amparo Gil, Luis M. Gutiérrez

**Affiliations:** ^1^Instituto de Neurociencias, Centro Mixto del Consejo Superior de Investigaciones Científicas, Universidad Miguel HernándezAlicante, Spain; ^2^Instituto Teófilo Hernando, Departamento de Farmacología y Terapéutica, Facultad de Medicina, Universidad Autónoma de MadridMadrid, Spain; ^3^Centro de Investigación Biomédica en Red de Enfermedades NeurodegenerativasAlicante, Spain; ^4^Department Matemática Aplicada y Ciencias de la Computación, Universidad de CantabriaSantander, Spain

**Keywords:** F-actin cytoskeleton, fodrin, vesicles, mitochondria, confocal microscopy, transmission electron microscopy

## Abstract

Cultured bovine chromaffin cells have been used extensively as a neuroendocrine model to study regulated secretion. In order to extend such experimental findings to the physiological situation, it is necessary to study mayor cellular structures affecting secretion in cultured cells with their counterparts present in the adrenomedullary tissue. F-actin concentrates in a peripheral ring in cultured cells, as witnessed by phalloidin–rodhamine labeling, while extends throughout the cytoplasm in native cells. This result is also confirmed when studying the localization of α-fodrin, a F-actin-associated protein. Furthermore, as a consequence of this redistribution of F-actin, we observed that chromaffin granules and mitochondria located into two different cortical and internal populations in cultured cells, whereas they are homogeneously distributed throughout the cytoplasm in the adrenomedullary tissue. Nevertheless, secretion from isolated cells and adrenal gland pieces is remarkably similar when measured by amperometry. Finally, we generate mathematical models to consider how the distribution of organelles affects the secretory kinetics of intact and cultured cells. Our results imply that we have to consider F-actin structural changes to interpret functional data obtained in cultured neuroendocrine cells.

## Introduction

Adrenomedullary chromaffin cells are widely used as a neuroendocrine model to study the exocytosis of dense core vesicles. The secretory process in these cells involves granule transport, the translocation of the secretory vesicles to the plasma membrane, their docking at secretory sites and finally, the fusion of the vesicle with the plasma membrane that results in the extrusion of their soluble contents (Burgoyne et al., 1993). Distinct cellular structures play fundamental roles in different stages of this secretory cascade, such as the cortical cytoskeleton (Trifaro et al., 2008; Gutierrez, 2012; Meunier and Gutierrez, 2016; Villanueva et al., 2016), while organelles like the mitochondria and endoplasmic reticulum appear to control and shape the elevations in intracellular calcium that govern multiple steps of this cascade (Garcia-Sancho and Verkhratsky, 2008; Garcia et al., 2012; Garcia-Sancho et al., 2012).

Interestingly, the vast majority of studies into secretory processes in chromaffin cells have been carried out on isolated and cultured cells (Livett, 1984), assuming that this “*in vitro*” model is a fair correlate of the “native” cells present in the adrenomedullary tissue. However, the aggregation of PC12 cells in culture enhances the release of catecholamines (Baldwin and Saltzman, 2001) and electrical coupling occurs among the cells that form part of the rat adrenomedullary gland (Moser, 1998), evidence of the importance of cell–cell contacts in shaping secretory responses. Indeed, it was proposed that the culture of whole adrenal tissue from rats could be a better physiological correlate of adrenomedullary cells than dissociated cells (Fujinaga et al., 1999).

To assess this postulate, we examined the state of the F-actin cytoskeleton and the distribution organelles like chromaffin granules and mitochondria in isolated cultured cells and in cells that form part of the bovine adrenomedullary tissue. Surprisingly, we found that the F-actin cytoskeleton clearly adopts a distinct distribution *in vitro* and *in situ*, which may affect the distribution of chromaffin granules and mitochondria. Since these organelles are essential to shape and regulate the neuroendocrine cell’s secretory behavior, our results stress the importance of the F-actin cytoskeleton in controlling the cells secretory responses. Accordingly, our data suggest that isolated and cultured cells may not be appropriate to study the true physiological characteristics of the secretory process. In addition, we have used mathematical models to understand how these structural changes between isolated and native cells might influence the secretory responses from both cellular conditions.

## Materials and Methods

### Chromaffin Cell Preparation and Culture

Chromaffin cells were isolated from bovine adrenal glands by collagenase digestion, and they were further separated from the cell debris and erythrocytes by centrifugation on Percoll gradients as described elsewhere (Gutierrez et al., 1989, 1995). The cells were maintained as monolayer cultures in Dulbecco’s modified Eagle’s medium (DMEM) supplemented with 10% fetal calf serum, 10 μM cytosine arabinoside, 10 μM 5-fluoro-2′-deoxyuridine, 50 IU/ml penicillin, and 50 μg/ml streptomycin. The cells were plated at a density of 150,000 cells/cm^2^ on 22 mm diameter poly-lysine coated coverslips. For the experiments, the cell culture media was replaced with basal Krebs/HEPES (K/H) solution containing: 134 mM NaCl, 4.7 mM KCl, 1.2 mM KH_2_PO_4_, 1.2 mM MgCl_2_, 2.5 mM CaCl_2_, 11 mM glucose, 0.56 mM ascorbic acid, and 15 mM HEPES [pH 7.4]. For microscopy experiments, cells were used between the third and sixth day after plating on glass coverslips (24 mm) in plastic Costar dishes (35 mm) at a density of 10^6^ cells/ml (2 ml per dish), performing all the experiments at room temperature (21–22°C). For catecholamine secretion experiments, cells were plated at a density of 10^6^ cells/ml in plastic Petri dishes (60 mm, 5 ml per dish).

### Preparation of Bovine Adrenomedullary Slices

In order to prepare the tissue slices, adrenal glands were dissected to separate the cortex from the medulla, and the medulla was then cut into small pieces and embedded in 4% low fusion agarose (Type VII-A, Sigma, Co., Madrid, Spain) to be cut by vibratome (Leica VT 1000). We obtained tissue slices (ca. 180 nm) at 6 Hz frequency from agarose cubes and all of them were submerged in BBS free calcium buffer (125 mM NaCl, 2.5 mM KCl, 10 mM MgCl_2_, 0.1 mM CaCl_2_, 26 mM NaHCO_3_, 1.25 mM NaH_2_PO_4_, 10 mM D-glucose) immediately after the cutting process. These slices were processed for immunohistochemistry the same day of collection as described below and then were maintained at 4°C. The slices were used for confocal microscopy on the second or third day after collection and all experiments were performed at room temperature (21–22°C).

### Confocal Microscopy of the F-Actin Cytoskeleton, α-Fodrin, and Chromaffin Granules

Cells and tissue slices were first fixed and then permeabilized using a modified method described previously (Lopez et al., 2007). Briefly, the samples were fixed in 4% paraformaldehyde (PFA) diluted in phosphate buffered saline solution (PBS) (20 min for cells and 30 min for tissue slices) and they were then permeabilized (10 min with 0.2% Triton X-100 in 3.6% formaldehyde for cells and 1 h with 0.5% Triton X-100 in 3.6% formaldehyde for tissue slices). The different application time was required in order to ensure a complete access of the antibodies to the cell interior. After that, in both cases the samples were washed twice with PBS for 15 min and twice with 1% BSA in PBS for 15 min.

The samples were then labeled to study the different cellular structures by confocal microscopy. F-actin structures were labeled with phalloidin coupled to rhodamine (Sigma-Aldrich, Madrid, catalog number P1951), at room temperature (21–22°C) during 45 min.

G-actin was labeled with a monoclonal antibody [1:200 dilution, Abcam Research Products, Cambridge, UK, Catalog number AB3280, Clon number ACTN05 (C4)]. The α-fodrin protein was labeled using a mouse anti-α-fodrin antibody (Abcam Research Products, Cambridge, UK, Catalog number AB131575, Clon number 3D7, Bath number GR196944-2). In addition, SNAP-25 was labeled with a polyclonal goat antibody (Santa Cruz Biotechnology, Texas, EEUU, Catalog number sc-7538, Bath number L0902). All primary antibodies were used at 1:200 dilutions in PBS.

Both G-actin and α-fodrin primary antibodies were visualized using a secondary antibody coupled to FITC (Anti-mouse to IgG produced in goat, Sigma Aldrich-Madrid, Catalog Number F-2012, Bach number SLBG3032). Two different secondary antibodies were used to visualize SNAP-25 labeling, one coupled to rhodamine (Anti-goat to IgG produced in rabbit, Santa Cruz Biotechnology, Texas, EEUU, Catalog number sc-3945) and the other to FITC (Anti-goat to IgG produced in donkey, Santa Cruz Biotechnology, Texas, EEUU, Catalog number sc-2024). All secondary antibodies were used at 1:200 dilutions in PBS.

All labeled cells and tissue slices were viewed on an Olympus Fluoview FV300 confocal laser system mounted on a IX-71 inverted microscope and using a 100X PLAN-Apo oil-immersion objective with 1.45 n.a. Excitation was achieved with Ar and HeNe visible light lasers.

### Transmission Electron Microscopy of Chromaffin Granules and Mitochondria

Bovine chromaffin cell pellets and adrenomedullary tissue (2–3 mm^3^) were fixed at 4°C with 2.5% glutaraldehyde in 0.2 M cacodylate buffer [pH 7.0] (2 h for cell pellets and 4 h for medulla tissue), and then washed overnight at 4°C in a solution of 0.2 M cacodylate buffer, sucrose and distilled water. After post-fixing at 4°C with 1% osmium tetroxide in 0.2 M cacodylate buffer (2 h for cell pellets and 4 h for adrenomedullary tissue) and extensive washing with distilled water, samples were stained at 4°C with 2% aqueous uranyl acetate (1 h for cell pellets and 2 h for adrenomedullary tissue). Subsequently, the samples were washed again, dehydrated through an ethanol series (30, 50, 70, 80, 96, and 99%: 15 min each one) and incubated twice for 15 min with propylene oxide at room temperature. Finally, the cell pellets and adrenomedullary pieces were embedded in epoxy resin and ultra-thin sections (70 nm) were obtained on a Leica UC6 ultramicrotome and transferred to copper grids (200 mesh). After staining with uranyl acetate for 5 min and lead citrate for 1 min, the ultrathin sections were analyzed on a JEOL 1011 a 80 kv transmission electron microscope with Gatan BioScam mod 792 digital camera to capture the images.

### Data Analysis from Microscopy Images

All microscopy images were analyzed with the Image J free software using the appropriate plug-ins to: automatically detect particles in thresholded images, calculate the area, perform density studies and intensity profile determinations, and perform 3D reconstructions. Graphics were prepared with IgorPro Graphpad Prism (GraphPad software, San Diego, CA, USA) and Adobe Photoshop 7.0. The non-parametric, the two-way ANOVA test was used to establish the significance of the experimental data (samples were considered significantly different when *P* < 0.05). The data were expressed as the mean + SEM from experiments performed on (n) individual cells, vesicles from at least two different cultures or adrenal tissue preparations.

### On-line Measurement of the Catecholamine Released by Native and Isolated Bovine Chromaffin Cells after Stimulation

To measure catecholamine release from intact isolated bovine chromaffin cell populations, cells were carefully recovered from the Petri dish using a rubber policeman and centrifuged at 800 rpm for 10 min. The cell pellet was resuspended in 200 μl of Krebs-HEPES (composition in mM: NaCl 144; KCl 5.9; CaCl_2_ 2; MgCl_2_ 1.2; glucose 11; HEPES 10 [pH 7.4]) and the cells were introduced into a microchamber for superfusion at the rate of 2 ml/min. To measure catecholamine release in adrenomedullary bovine tissue, small pieces of tissue (ca. 5–8 mm^3^) were obtained from adrenal glands and introduced into a microchamber for superfusion with Krebs-HEPES at the rate of 2 ml/min.

The microchamber had a volume of 100 μl and it was covered with a jacket to continuously circulate external water at 37°C. To detect the catecholamines released, the liquid flowed from the superfusion chamber to an electrochemical detector (Metrohn AG CH-9100 Herisau, Switzerland) equipped with a glassy carbon working electrode, an Ag/AgCl reference electrode and a gold auxiliary electrode. Catecholamines were oxidized at +0.65 V and the oxidation current was recorded on line by a PC placed at the outlet of the microchamber under the amperometric mode, assessing the amount of catecholamines secreted (Borges et al., 1986). Secretion was stimulated to with 5 s pulses of a Krebs-HEPES solution containing 100 μM Acetylcholine (ACh) and the solutions were rapidly exchanged through electrovalves driven by a PC.

### Modeling the Effect of Granule and Mitochondrial Organization on Chromaffin Cell Secretion

To simulate secretory events we used a Monte Carlo algorithm that proved to be successful in the study of calcium buffered diffusion (Gil et al., 2000), of the influence of geometrical factors on the exocytotic response of neuroendocrine cells (Segura et al., 2000; Torregrosa-Hetland et al., 2011) and of presynaptic terminals (Gil and Gonzalez-Velez, 2010). The algorithm implements a microscopic simulation in which the fundamental variables are the number of ions and buffers. The average values of the output of our simulations converge to macroscopic results when considering symmetric configurations.

Calcium-induced secretory events in the sub-membrane domain of spherical cells (as is the case of chromaffin cells in close approximation) can be adequately described using a conical subdomain where the different processes involved take place: calcium entry through voltage-dependent calcium channels (VDCCs); the kinetic reactions of calcium and buffers; the diffusion of mobile buffers and calcium ions; and the binding of calcium ions to secretory granules. The base of the cone represents the membrane of the cell where calcium channels cluster. We consider these clusters to be formed by two P/Q- and one L-type calcium channels, according to experimental estimations of channel populations involved in chromaffin cell secretion (Lukyanetz and Neher, 1999). A schematic representation of the 3-D simulation domain is shown in **Figure 8A**, in which three clusters of VDCCs and a few mitochondria are also represented. The simulation of currents through these channel types is made using a simple stochastic scheme where every channel of the total population can transit from its present state to an open, closed or inactive state in response to voltage and calcium concentrations. The current to voltage relationships considered in the channel gating kinetic schemes for P/Q- and L-type calcium channels are shown in **Figure 8B**.

The conical domain is mapped by a 3-D regular orthogonal grid with a distance between grid points Δx. The 3-D diffusion of mobile particles in the simulation domain is modeled as a random walk process. The first-order kinetic reactions of calcium ions and buffers are interpreted and resolved probabilistically. This also applies to the binding of calcium ions to secretory granules which are, in this sense, interpreted as an additional calcium buffer in the medium. The kinetic model of Klingauf and Neher (1997) for the secretory sensor of vesicles in chromaffin cells is used in the algorithm.

Apart from granules, mitochondria are also considered in the simulation domain. The mitochondria and secretory granules inside the simulation domain are distributed based on experimental findings from isolated cells and cells in adrenal slices. Mitochondria are modeled as permeable obstacles to diffusion and when calcium ions are trapped in the diffusion process by a compartment corresponding to mitochondria, the obstructed diffusion inside the mitochondrial matrix is simulated by moving them in exactly the same way that calcium ions are moved in the cytoplasm, but only after a number “N” of simulation steps. The waiting time in the mitochondrial matrix can be estimated by taken into account experimental evidence from chromaffin cells that suggests the amount of calcium taken up by the subpopulation of mitochondria in the immediate vicinity of the VDCC clusters can be very large during the maximal stimulation of calcium entry through calcium channels, in the order of a few 100 μM (Villalobos et al., 2002).

### Ethics Statement

Adrenal glands were obtained from an industrial slaughterhouse (Matadero de Orihuela SA) subjected to strict regulations of the Ministries of Agriculture, Industry and Health of Spain in accordance with EC normative.

All the protocols described in this article were approved by the Organo Evaluador de Proyecto of University Miguel Hernández, the office in charge of the observation of the Ethics in animal care and experimentation in our institution.

## Results

### The F-Actin Cytoskeleton Accumulates in the Cortical Area of Isolated Cells whereas it Is Distributed Evenly throughout the Cells in the Tissue

**FIGURE 1:**
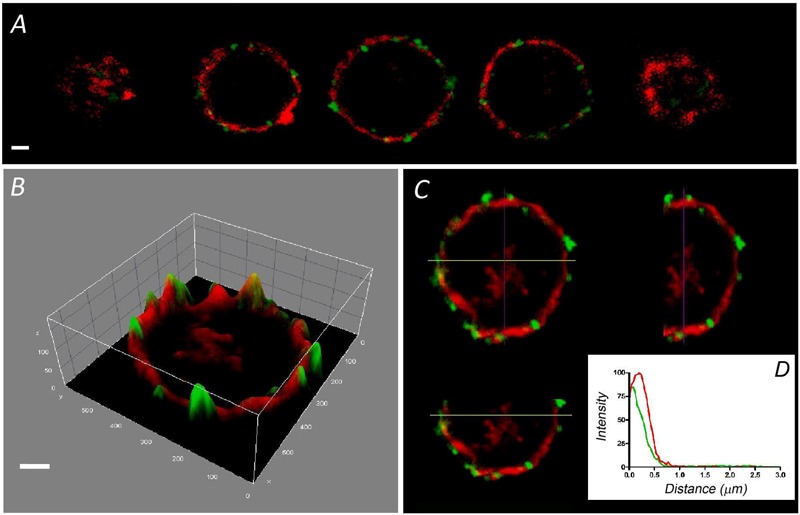
**Distribution of F-actin in cultured bovine chromaffin cells.** Sequential confocal images of a representative chromaffin cell in culture labeled with rhodamine–phalloidin (red fluorescence), and anti-SNAP 25 in green **(A)**. **(B)** The intensity distribution of the central image of **(A)** is shown depicting the concentration of fluorescence at the cell periphery. A pseudo-3 D reconstruction highlights the peripheral labeling present in the three spatial axis **(C)**. **(D)** The individual fluorescence profiles were averaged to obtain the distribution of F-actin fluorescence relative to the position of the plasma membrane labeled with anti-SNAP-25 (green). Data from cultured cells (*n* = 16 cells, two different cell preparations). Bars in **(A,B)** represent 1 μm.

**FIGURE 2:**
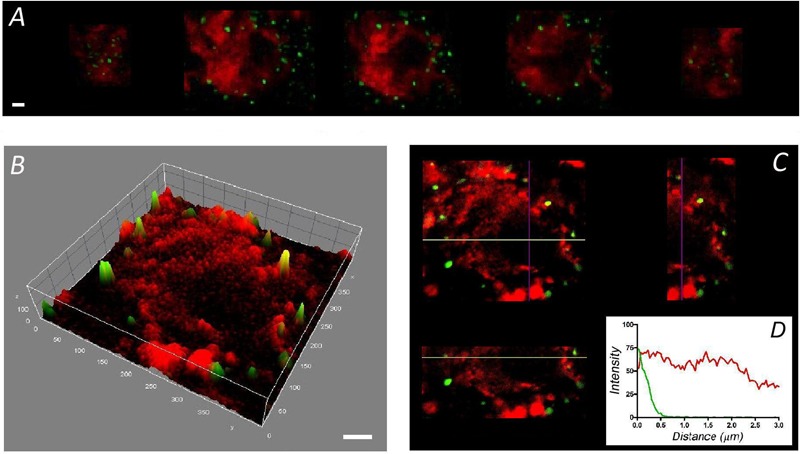
**Distribution of F-actin in chromaffin cells from adrenal medulla slices.** Sequential confocal images of cells in slices of adrenomedullary tissue labeled with rhodamine–phalloidin (red) and anti-SNAP-25 (green) **(A)** showing a broader cytoplasmic distribution of F-actin, as confirmed by the intensity study of the central image **(B)**, and the spatial plane analysis **(C)**. **(D)** The individual fluorescence profiles were averaged to obtain the distribution of F-actin fluorescence (red line) relative to the plasma membrane position (green line) of cells in the adrenal medulla (*n* = 16 cells, two different tissue preparations). Bars in **(A,B)** represent 1 μm.

The F-actin cortical cytoskeleton has been characterized extensively in cultured bovine chromaffin cells as a factor that strongly influences secretory behavior in this neuroendocrine model (Trifaro et al., 2008; Gutierrez, 2012; Meunier and Gutierrez, 2016). Interestingly, few such studies have been performed on the native cells that form part of the adrenal medulla. Therefore, we set out to compare the distribution of this essential structural element in dissociated and cultured cells with that in bovine adrenomedullary slices by confocal fluorescent microscopy. Rhodamine–phalloidin clear labeled the cortical F-actin in fixed and permeabilized cultured cells as described in a variety of earlier studies. The sequence of confocal planes from a representative cell revealed the accumulation of fluorescence in the cortical region forming a peripheral ring (**Figure 1A**), as also evident after fluorescence integration studies (**Figure 1B**) and in the analysis of the different spatial axis (**Figure 1C**). In these images the position of the cell membrane was visualized using an antibody against SNAP-25 (green fluorescence in **Figure 1**). Moreover, after averaging the fluorescent profiles for many cells corresponding to two different cell cultures (*n* = 16 cells, **Figure 1D**), the virtual absence of internal F-actin is notable. Interestingly, the distribution of F-actin in cells present in adrenomedullary slices is quite different (**Figure 2**) with phalloidin staining distributed in patches not only in the cortical region but also throughout the cytoplasm. The presence of these patches is clear in fluorescence intensity studies (**Figure 2B**) and throughout the different spatial planes (**Figure 2C**). Hence, averaging a high number of these profiles (*n* = 16) indicates that sustained levels of F-actin are distributed throughout the cytoplasmic space (**Figure 1D**).

**FIGURE 3:**
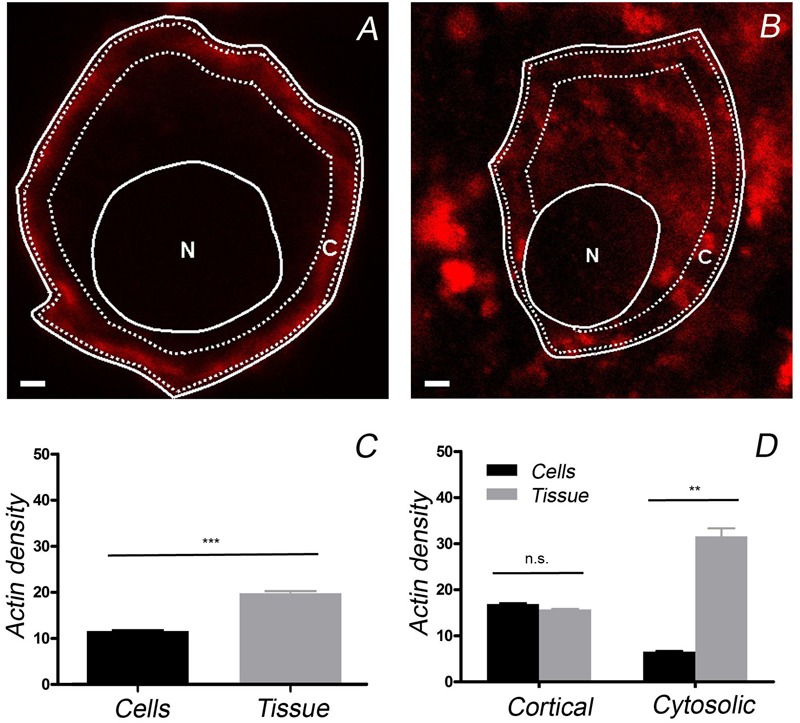
**Quantification of the F-actin density in cultured chromaffin cells and in cells present in the adrenal medulla.** Confocal images of F-actin labeled with rhodamine–phalloidin were used to calculate the F-actin density in the whole cell and in the cortical region (“C”), as well as in the cytoplasm (excluding the nuclear zone, “N”). The average F-actin density appeared to be higher in the cells forming part of the adrenomedullary tissue **(C)**, mainly due to the presence of abundant cytoplasmic F-actin in the cells present in this tissue **(D)**. A similar density of cortical F-actin is evident in cultured cells (*n* = 20 cells) and those forming part of the adrenomedullary tissue (*n* = 20 cells). Bars in **(A,B)** represent 1 μm. ^∗∗^*P* < 0.005, ^∗∗∗^*P* < 0.0005 using the two-way ANOVA test.

To calculate the F-actin density in different regions of the cells studied, the fluorescence intensity was calculated for the cortical region expanding 1 μm from the cell’s periphery and divided by the area occupied (**C** in **Figures 3A,B**). Similarly, the F-actin fluorescence density in the cell interior was calculated, excluding the nuclear region (**N** in **Figures 3A,B**). After averaging the fluorescence density in the cell area excluding the nucleus, it was evident that the density of F-actin was lower in cultured chromaffin cells (*n* = 20, from three cell preparations) when compared with that in adrenomedullary cells (*n* = 20, from three adrenal tissue preparations) (**Figure 3C**). This is due to the more extended distribution of cytoplasmic F-actin in native cells as the density of cortical F-actin was similar in both cell types (**Figure 3D**).

To address whether the absence of cytosplasmic F-actin in cultured cells was due to a lack of g-actin for polymerization, the distribution of g-actin was evaluated using specific antibodies (Supplementary Figure S1). There was considerable g-actin present in the cytoplasm of cultured cells that is not polymerized, whereas in cells within the adrenomedullary tissue the g-actin co-localized with F-actin, indicating significant polymerization of actin throughout the cytoplasm.

This difference in the distribution of F-actin could not be attributed to the absence of cell to cell contacts or the limited access of phalloidin in cultured cells since increasing the density of cells in cell cultures or prolonging the experimental time of incubation did not affect the F-actin distribution (Supplementary Figure S2).

### Differential Distribution of α-Fodrin between Isolated Adrenal Cells and Those Present in the Adrenal Gland

**FIGURE 4:**
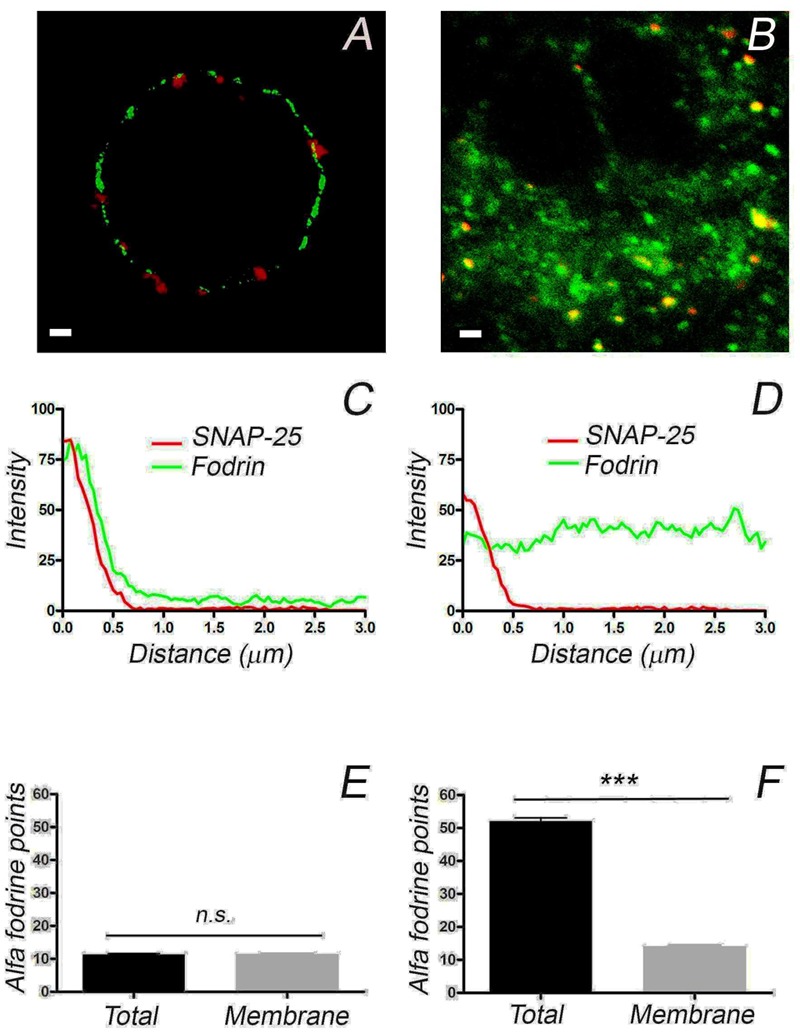
**The distribution of α-fodrin in cultured cells and cells from adrenomedullary slices.** Confocal images of α-fodrin (green fluorescence) and anti-SNAP-25 (red fluorescence) used to calculate the density of α-fodrin in the whole cell and at the plasma membrane (Membrane), as indicated in the previous Figure. Individual density profiles were used to obtain the averaged α-fodrin profiles from cultured cells (**C**, *n* = 11, 2 cell preparations) and in cells from adrenomedullary slices (**D**, *n* = 16, 2 adrenal medulla preparations). The average total α-fodrin patches appear to be much higher in the cells forming part of the adrenomedullary tissue **(F)**, this being due to the abundant cytoplasmic α-fodrin. There are a similar number of membrane associated α-fodrin patches in cultured cells and cells from the adrenomedullary tissue **(E,F)**. Bars in **(A,B)** represent 1 μm. ^∗∗∗^*P* < 0.0001 using the two-way ANOVA test.

The differences in the distribution of F-actin in cultured and native cells present in the adrenomedullary tissue was an unexpected finding that could affect the distribution of other proteins and organelles, thereby influencing the secretory behavior of these cells. The protein α-fodrin associates with both F-actin and the plasma membrane, and it becomes redistributed during secretion (Perrin and Aunis, 1985; Langley et al., 1986; Burgoyne and Cheek, 1987; Perrin et al., 1987). Therefore, we assessed whether the distribution of this protein also differed in these cells, in line with the changes observed for F-actin. Using a mouse monoclonal antibody against α-fodrin, a punctate distribution of this protein that was clearly associated with the cell periphery was evident in confocal fluorescence microscopy images of cultured cells (**Figure 4A**), while its was more extensively distributed throughout the cytoplasm in the cells forming part of the adrenomedullary tissue (**Figure 4B**). Again, the intensity profiles from individual cells and profile averaging confirmed the broad cytoplasmic distribution of α-fodrin in the intact tissue (**Figure 4D**) (*n* = 16), in contrast to its concentration at the cortex of cultured cells (**Figure 4C**) (*n* = 11). Interestingly, the number of peripheral α-fodrin puncta was very similar at the periphery of both cell types but obviously, there was a considerable number of cytoplasmic α-fodrin patches in the tissue that were absent in cultured cells (**Figures 4E,F**). Therefore, it seems clear that both cultured cells and those forming part of the intact adrenomedullary tissue have a very similar cortical density of F-actin and α-fodrin, whereas there is a dense cytoplasmic F-actin structure in the latter to which intracellular α-fodrin associates.

### The Distribution of Chromaffin Granules and Mitochondria Differs in the Adrenal Gland and in Cultured Cells

**FIGURE 5:**
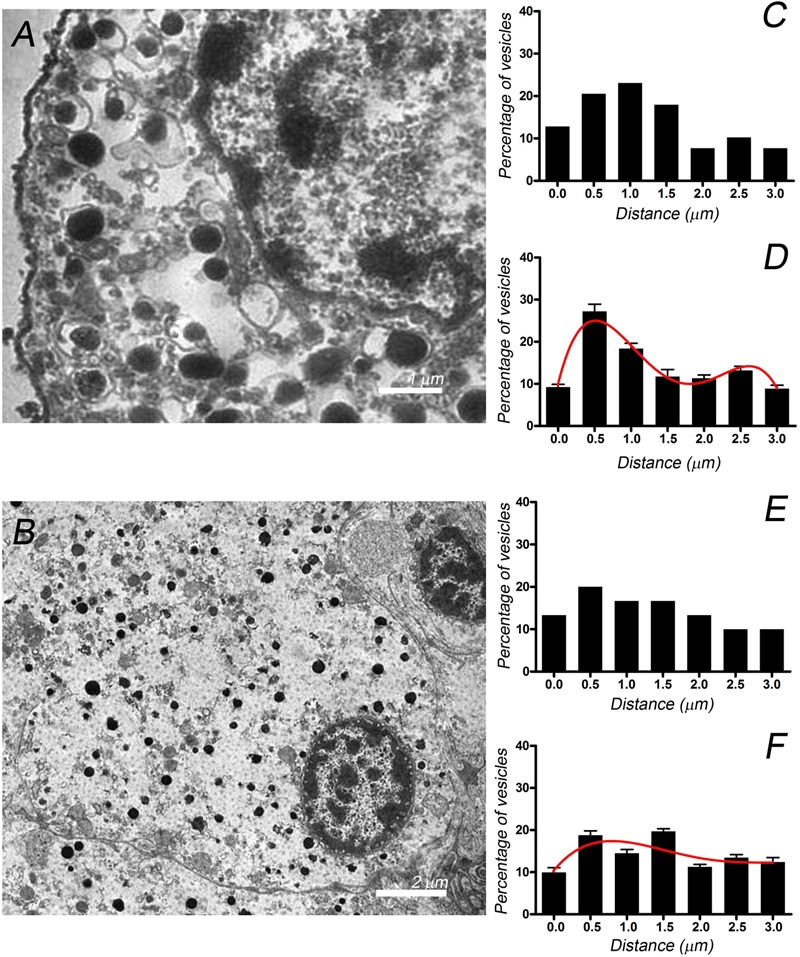
**Chromaffin granule distribution studied by EM in isolated cells and cells present in the adrenal medulla.** EM micrographs corresponding to individual cultured cells **(A)** and cells present in the adrenomedullary slices **(B)**. These images were used to calculate the granule position with respect to the cell membrane, generating the granule distributions depicted for the cultured cell sampled **(C)** and the cell in the tissue **(E)**. Vesicle distributions were used to obtain the averaged granule distributions for cultured cells (**D**, *n* = 10 cells, 308 vesicles) and cells in the adrenal tissue (**F**, *n* = 10 cells, 302 vesicles). The curves in **(D,F)** correspond to the best fit to a binomial distribution. Bars in **(A,B)** represent 2 and 5 μm, respectively.

To examine whether or not the different distributions of F-actin in the isolated and native cells influences the distribution of organelles essential for secretion, we studied the distribution of chromaffin granules by electron microscopy (**Figures 5A,B**). The position of dense chromaffin granules was obtained by manual distance measurements of vesicles located in the studied section delimited from the plasma membrane limit to 3 μm inside cytoplasm and used to build granular distributions for individual cells (**Figures 5C,E**). Mean values of granules distribution (**Figures 5D,F**) were obtained by averaging corresponding data from 10 isolated cells (*n* = 308 vesicular distances, two cell preparations) and 10 “native” cells present in the adrenal tissue (*n* = 302 vesicular distances, two adrenal tissue preparations). Interestingly, chromaffin granules seem to present a biphasic distribution in the isolated cells with two different cortical and internal populations, whereas their distribution was more homogeneous in the cells present in the adrenal medulla. Furthermore, in an additional experiment the treatment of the cells with latrunculin A, altering F-actin organization in our cells (Giner et al., 2005), caused the redistribution of the vesicles resulting in the disappearance of the biphasic distribution (Supplementary Figure S3).

In parallel, the differential distribution of chromaffin vesicles between the cultured and the cells present in the adrenal medulla has been supported also by experiments performed using chromaffin granule labeling with anti-Dopamine β-Hidroxylase (Supplementary Figure S4).

**FIGURE 6:**
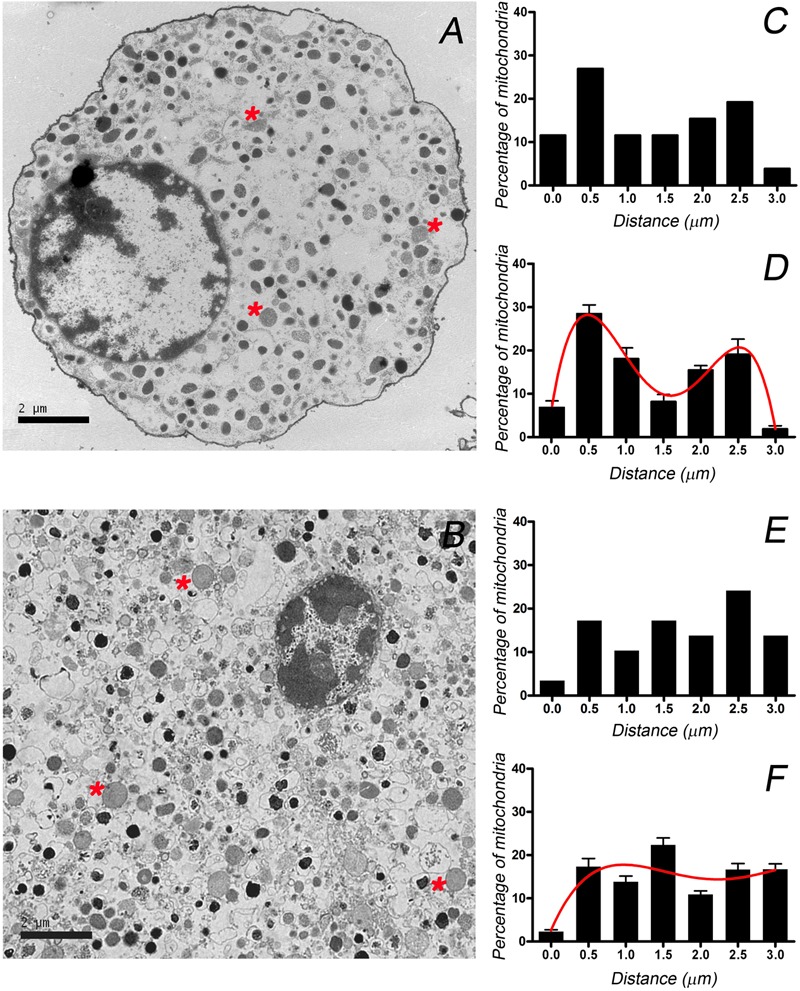
**Distribution of mitochondria from cultured and bovine chromaffin cells present in the adrenal medulla.** Mitochondria (asterisks) were identified in EM micrographs and their distribution was studied in individual cultured cells **(A)** and in cells in adrenomedullary slices **(B)**. These images were used to calculate the position of the mitochondria with respect to the cell membrane in order to generate a distribution of mitochondria as depicted for a sample cultured cell **(C)** and one in the tissue **(E)**. Mitochondrial distributions were used to obtain the averaged distributions for cultured cells (**D**, *n* = 12 and 224 mitochondria) and cells in the adrenal tissue (**F**, *n* = 10 cells and 247 mitochondria). Curves in **(D,F)** correspond to the best fit to binomial distributions. Bars in **(A,B)** represent 2 μm.

The differential distribution of granules between the cultured cells and those forming part of the tissue could reflect how the distinct cytoskeletal organization influences the distribution of other major organelles. Thus, we studied the distribution of mitochondria in electron microscopy images since individual mitochondria can be clearly distinguished in such images by their shape, lower density and internal cristae (**Figure 6**, see asterisks). Again, the distance of the mitochondria to the cell membrane was used to define their distribution in individual cells (**Figures 6C,E**) and averaged to obtain the mean distributions (**Figures 6D,F**) for cultured (*n* = 10) and the cells present in the adrenal tissue (*n* = 10). The resulting distributions again sustain the notion that mitochondria present a bimodal distribution in isolated cells indicating the presence of distinct cortical and internal populations, whereas they were more homogenously distributed in the native cells in the medullary tissue. The results obtained for cultured cells are in agreement with recent confocal microscopy studies (Villanueva et al., 2014), that in addition proved that the alteration of F-actin organization caused the redistribution of mitochondria with the subsequent disappearance of the biphasic distribution.

Therefore, our results strongly suggest that the F-actin distribution in the native and isolated cells is responsible for the differences in the distribution of major organelles like chromaffin granules and mitochondria, potentially affecting the secretory behavior of chromaffin cells.

### Secretory Behavior of Native and Isolated Bovine Chromaffin Cells

**FIGURE 7:**
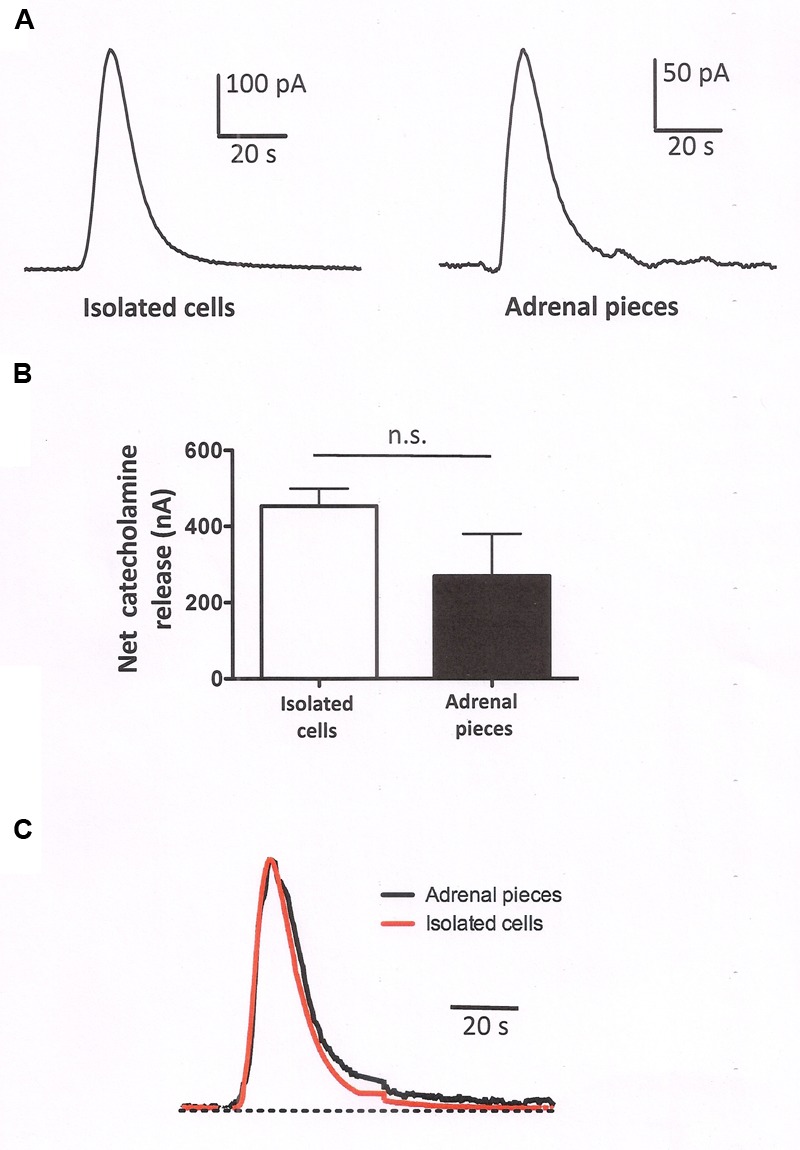
**Secretory response from populations of isolated packed cells and pieces of adrenomedullary tissues.** Catecholamine release from populations of chromaffin cells measured amperometrically upon stimulation of the cells with 5 s pulses with a Krebs-HEPES solution containing 100 μM ACh. **(A)** Show representative amperometric recordings obtained from packed isolated cells or from adrenal tissue pieces as indicated in methods. **(B)** Shows the averaged catecholamine release from isolated cells versus the release obtained in adrenal tissue. The data are the means ± SEM of nine experiments from three different preparations. **(C)** The averaged catecholamine release has been normalized to the maximal release in order to show that there are no changes in the onset kinetics and only a small change in the offset kinetics when the responses obtained in isolated cells were compared with those from adrenal tissue.

To assess whether the secretory response of isolated cells and cells forming part of the native adrenal tissue differs, we evaluated the catecholamine release from both cell types using an amperometric approach using populations of packed cells. Isolated chromaffin cells (5 × 10^6^ cells) were packed in a microchamber and their secretory response examined when stimulated by acetylcholine (Ach, 100 μM), the physiological neurotransmitter at the splanchnic nerve-chromaffin synapse (**Figure 7A**). The secretory responses of the isolated cells were compared with those obtained from small pieces (five pieces of about 5–8 mm^3^) of the native adrenal gland after exhaustive rinsing with a basal Krebs-HEPES solution. When measured by amperometry (*n* = 9 experiments), the responses in the isolated cells were of a higher magnitude (**Figure 7B**) but when normalized to the maximal value, these responses were remarkably similar in terms of the onset kinetics yet with a slightly slower offset kinetics in the intact cells (**Figure 7C**). Therefore, it appears that the kinetics of catecholamine release do not differ greatly in isolated cells and in cells forming part of the adrenal gland, despite the important changes in cytoskeletal organization and organelle distribution described above, and in any case, a small change in the offset kinetics could be associated with a more sustained response in native cells and this might be related with the presence of slow releasable internal vesicle pools.

## Discussion

### The Internal F-Actin Cytoskeleton Is Destabilized When Cells Are Isolated from the Adrenal Gland

Cultured chromaffin cells have been used extensively as a secretory model to investigate the molecular basis of the exo-endocytosis cycle (Burgoyne, 1995; Trifaro et al., 2008; Gutierrez, 2012; Papadopulos et al., 2013; Meunier and Gutierrez, 2016). However, in cultured cells the distribution of calcium channels and SNARE proteins may be altered with respect to that in native cells (Lopez et al., 2007), which may affect the functional properties of the cells when isolated from a tissue like the adrenal medulla. The data presented here shows that the F-actin cytoskeleton, the major structural element supporting cell shape, organelle transport (Villanueva et al., 2016) and an essential element of the secretory machinery (Gutierrez, 2012; Papadopulos et al., 2013; Meunier and Gutierrez, 2016), is also altered dramatically after isolation and culture. These changes are not surprising since cell-to-cell contacts and cell adhesion exert a significant influence on the cytoskeleton (Kaibuchi, 1999; Braga, 2002).

### Essential Proteins Associated with F-Actin and Organelles Are Distributed Distinctly in Isolated Cells and Chromaffin Cells in the Adrenomedullary Tissue

The differences in F-actin distribution between isolated and native chromaffin cells, were also reflected in the localization of the fodrin protein, a protein that associates with F-actin. The interaction between F-actin and fodrin was first reported on the basis of their co-sedimentation in enriched fractions (Levine and Willard, 1981), and fodrin was characterized as a cortical protein, “lining” the cell membrane in neural and non-neural tissues as reflected in its name (from “fodros,” lining in Greek). The α subunit of fodrin was then shown to bind to F-actin in the submembrane space, which was also responsible for its reorganization during cell stimulation (Perrin and Aunis, 1985). Today, α-fodrin is considered as a cytoskeletal-related protein (Haneji et al., 1997), and it has been associated with other relevant exocytotic proteins like syntaxin 1, 3 and 4, forming part of the fusion pore (Nakano et al., 2001; Jena et al., 2003). The spatial distribution of α-fodrin in isolated and native chromaffin cells defined here confirms its association with F-actin not only in the submembrane domain, as described previously, but also in important cytoplasmic domains in native chromaffin cells. Given the relationship between both these elements, the common spatial distribution confirms our data regarding the differences in F-actin distribution, raising questions regarding the possible role of the association of α-fodrin with F-actin in the cell interior of native cells.

An important consequence of the distinct F-actin organization in cultured and native chromaffin cells is the altered localization of organelles like chromaffin granules and mitochondria. In this context the cortical F-actin structure in neuroendocrine cells was seen to act as a system to “anchor” chromaffin granules (Oheim and Stuhmer, 2000; Johns et al., 2001) and mitochondria (Villanueva et al., 2014) in the vicinity of active sites. Therefore, it is not a surprise that there is a dense meshwork of F-actin in the interior of the native cells described here that is absent from isolated and cultured cells. This F-actin network helps to stabilize a larger population of internal organelles and consequently, alter their cellular distribution.

### Functional Models Analyzing the Impact of Organelle Distribution in the Secretory Responses between the Intact and Isolated Chromaffin Cells

**FIGURE 8:**
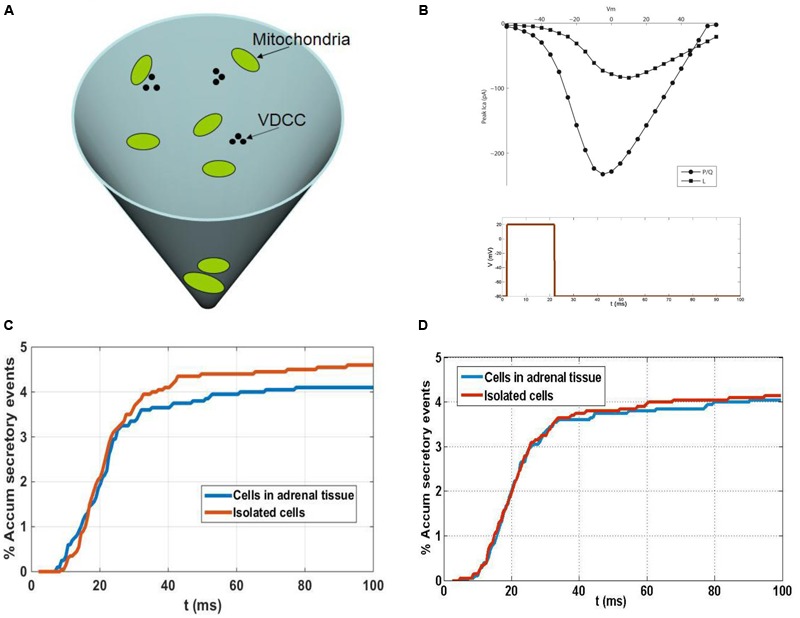
**A theoretical model to understand the influence of granule and mitochondria in secretion from cultured and native chromaffin cells. (A)** Schematic representation of the 3-D simulation. **(B)** Upper figure: current to voltage relationships considered in the channel gating kinetic schemes for P/Q- and L-type calcium channels. Lower figure: depolarizing pulse considered in the simulations. **(C)** Comparison of secretory responses predicted by the model in the absence of mitochondria: theoretical accumulated secretory responses (percentage) for isolated cells and cells in adrenal tissue obtained using the experimental granule distributions. No mitochondria are considered in the medium. **(D)** Comparison of secretory responses predicted by the model in the presence of mitochondria: theoretical accumulated secretory responses (percentage) for isolated cells and cells in adrenal tissue obtained using the experimental granule and mitochondria distributions.

It is important to consider what possible functional impact the distinct distribution of F-actin and organelles might have on cultured and native cells. We addressed this issue by using mathematical models that have proven useful to understand aspects of the secretory process in chromaffin cells (Segura et al., 2000; Torregrosa-Hetland et al., 2011). The accumulated secretory response (as a percentage) was defined by our model for isolated cells and cells in adrenal tissue (**Figure 8C**), simulating the response to a depolarizing pulse (**Figure 8B**). No mitochondria were considered in the medium in these simulations and the results shown are the average of a few 100 random configurations of the distances of vesicles from the VDCC. Random configurations were generated according to the experimental granule distributions summarized (**Figure 8D**) and as a result, the time courses of the secretory responses were very similar during the first 25 ms (**Figure 8C**). This result was consistent with the fact that the sub-population of vesicles that are released first are those that are in very close proximity to the VDCC, and that the proportion of these vesicles is nearly the same in isolated cells and in native cells in adrenal tissue (10% of the whole population of secretory granules in both cases). After the first 25 ms, a second sub-pool of vesicles situated further away from the VDCC come into play, a sub-pool of granules that represents approximately 27% of the granules in individual cells and approximately19% in the native cells in adrenal tissue (**Figure 8D**). Due to the larger size of this sub-pool of vesicles in isolated cells, a larger secretory response would be expected when the calcium concentration in the vicinity of these vesicles surpasses the threshold level needed to trigger release relative to that in the native cells in adrenal tissue (**Figure 8C**). However, this situation changes when mitochondria are considered in the medium, as the larger population of mitochondria close to the cell membrane in isolated cells than in the native cells in adrenal tissue (**Figure 8D**) act as a more effective barrier for the diffusion of calcium from the VDCC toward other parts of the cytoplasm, and in particular toward the location of the second sub-pool of granules. As a result, a larger reduction in the secretory response would be expected in isolated cells than in cells in adrenal tissue. The combined effects on vesicles and mitochondria, acting in opposite directions, influences the release of granules (as shown in **Figure 8D**), although the results obtained with our model indicate that one effect could actually compensate for the other. As a result, the secretory time courses in isolated cells and in native cells in adrenal tissue would look similar despite the differences in the distribution of their granules and mitochondria. This theoretical result is consistent with the results showing similar secretory behaviors of the two cell populations studied in our amperometric experiments (**Figure 7**), although the temporal resolution of this technique studying the release of 1000s of cells is relatively limited. In this sense, we should mention that ours and other lab efforts to measure secretion from individual cells forming part of the bovine adrenal medulla has been unsuccessful (Drs Antonio G. Garcia and Ricardo Borges personal communication).

### Thus, Are Isolated and Cultured Neuroendocrine Chromaffin Cells an Inadequate Model to Study Secretion?

The distinct distribution of F-actin between isolated and native chromaffin cells is relevant given the importance of this structure as a decisive organizer of the functional configuration of the exocytotic machinery. Differences in cytoskeletal organization alter the spatial distribution of proteins (e.g., α-fodrin), and of organelles and vesicles, which could be relevant when considering if isolated chromaffin cells are an appropriate model to study secretion. Although cultured chromaffin cells display such differences in an element that strongly influences the configuration of the secretory apparatus, and consequently they do not fully reflect the true physiological system, they do maintain the native level of cortical F-actin in a similar fashion as in native cells. The presence of this conserved peripheral cytoskeletal network probably means that isolated cells are indeed a reliable model to study membrane-related processes such as secretion but not all aspects of chromaffin cell biology. In these sense, for example, the study of organelle transport may differ clearly when comparing the situation of a low F-actin density cytoplasm found in cultured cells with the really structured network characteristic of native cell cytoplasm. Therefore, the development of strategies allowing working with native cells appears essential in order to fully understand the “physiological” transport and secretory machineries in neuroendocrine models.

## Author Contributions

YG-M, JV, CN, IL-F, MMF, performed and analyzed the experiments, YG-M, JV, SV, LG, and LMG designed the experiments, AG performed the model simulations, and LG, AG, and LMG wrote, reviewed and edited the manuscript. All authors approved the final manuscript.

## Conflict of Interest Statement

The authors declare that the research was conducted in the absence of any commercial or financial relationships that could be construed as a potential conflict of interest.
