# Supplementary material for: The Differential Organization of F-Actin Alters the Distribution of Organelles in Cultured When Compared to Native Chromaffin Cells
Source: Front Cell Neurosci. 2017 May 4;11:135. doi: 10.3389/fncel.2017.00135 (PMC5415619; doi:10.3389/fncel.2017.00135)
Supplement: Supplementary file 1 [file Image_1.PDF]

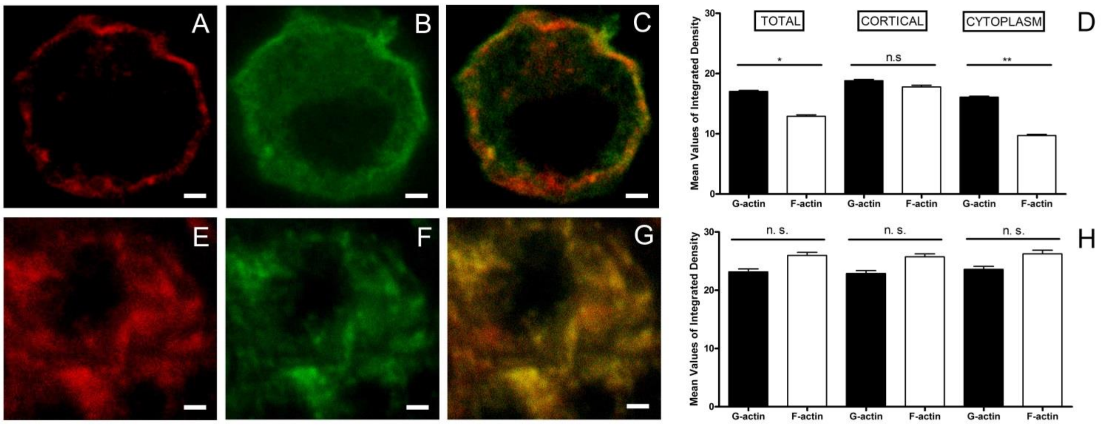

Fig. S 1. Comparative levels of F and G actin densities in cultured chromaffin cells and in cells present in the adrenal medulla. Confocal images of F-actin labelled with rhodamine-phalloidin (red) and G-actin labelled with a monoclonal antibody (green) were used to calculate the F and G-actin density in different regions of cultured (A-C), and native cells (E-G). Overlapping images were shown in yellow (C and G). The average F-actin density appeared to be higher in the cells forming part of the adrenomedullary tissue, mainly due to the presence of abundant cytoplasmic F-actin in the cells present in this tissue (D, ** $P<0.005$ compare to the value obtained for the cortical region). A similar density of cortical F-actin is evident in cultured cells (n=20 cells) and those forming part of the adrenomedullary tissue (n=20 cells). Bars in A and B represent 1 μm.* $P<0.05$ ** $P<0.01$.
