# Supplementary material for: The Differential Organization of F-Actin Alters the Distribution of Organelles in Cultured When Compared to Native Chromaffin Cells
Source: Front Cell Neurosci. 2017 May 4;11:135. doi: 10.3389/fncel.2017.00135 (PMC5415619; doi:10.3389/fncel.2017.00135)
Supplement: Supplementary file 2 [file Image_2.PDF]

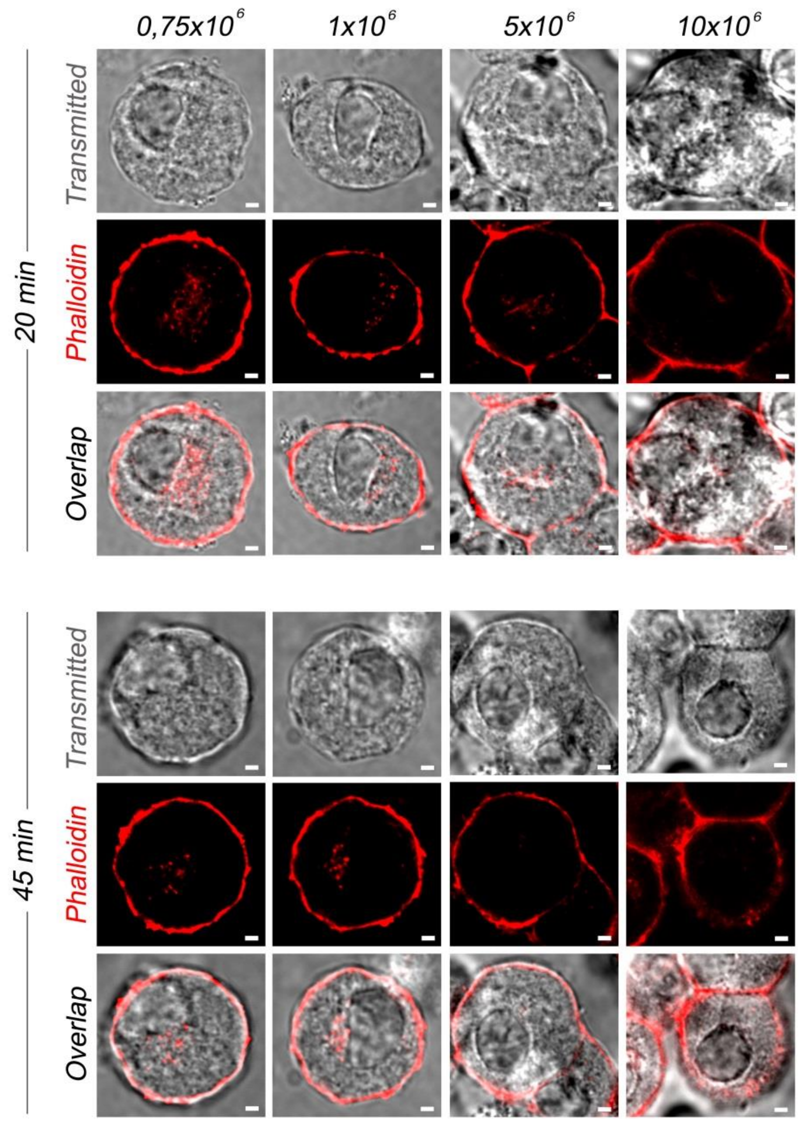

Fig. S 2. Changing fixation time and culture cell density do not affect peripheral F-actin labelling.. Confocal images of F-actin labelled with rhodamine-phalloidin (red) from chromaffin cell plated at different densities (on top is indicated the number of cells plated in 35 mm culture dish). During these experiments we tested also the influence of prolonging cell fixation from 20 min to 45 min to compare with the conditions used for labeling F-actin in adrenal slices. Bars represent 1 µm.
