# Supplementary material for: The Differential Organization of F-Actin Alters the Distribution of Organelles in Cultured When Compared to Native Chromaffin Cells
Source: Front Cell Neurosci. 2017 May 4;11:135. doi: 10.3389/fncel.2017.00135 (PMC5415619; doi:10.3389/fncel.2017.00135)
Supplement: Supplementary file 3 [file Image_3.PDF]

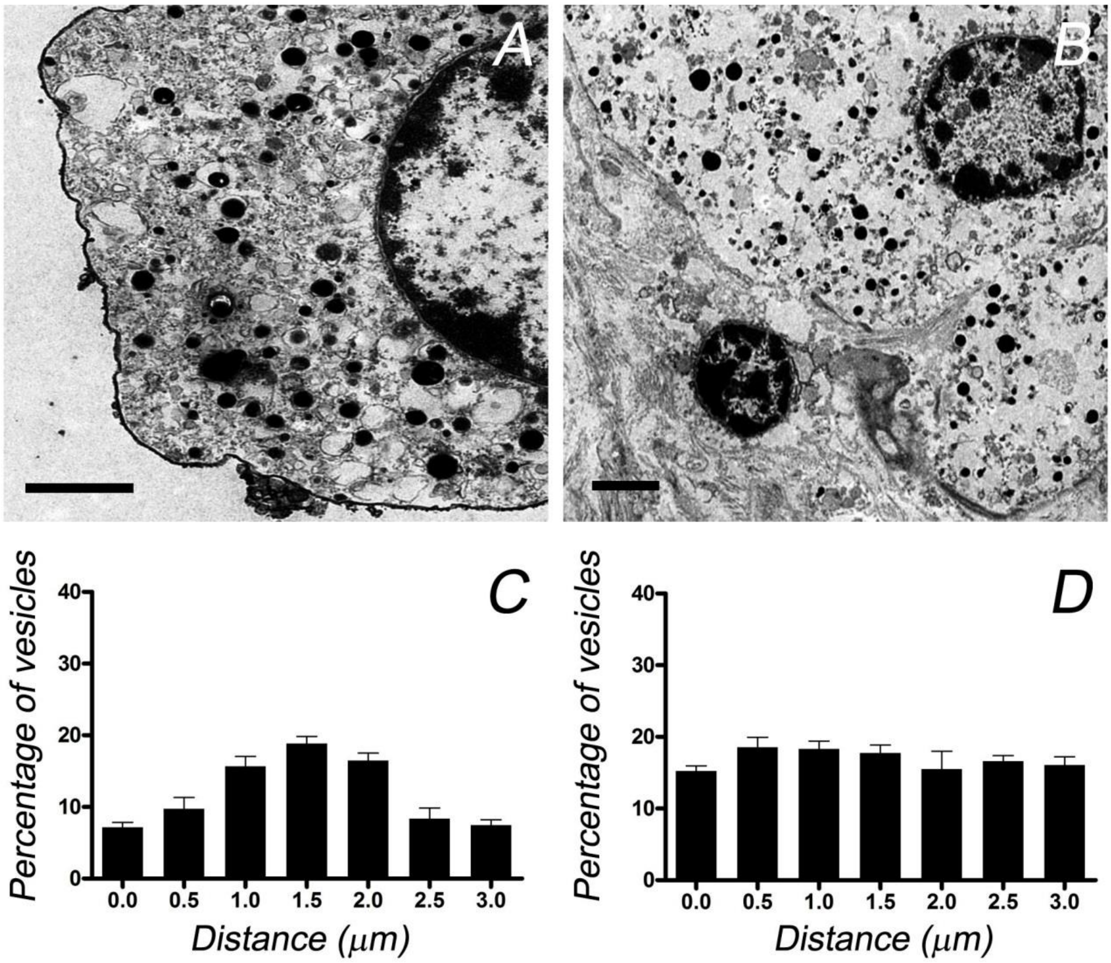

Fig S 3. Latrunculin A treatment alters the distribution of chromaffin granules in cells in culture.

EM micrographs corresponding to individual cultured cells (A) and cells present in the adrenal slices treated with 5 µM latrunculin A during 1 h. Vesicle distributions were used to obtain the averaged granule distributions for treated cultured cells (C, n=6 cells, 202 vesicles) and treated cells in the adrenal tissue (D, n=6 cells, 208 vesicles). Bars in A and B represent 1 µm.
