# Supplementary material for: The Differential Organization of F-Actin Alters the Distribution of Organelles in Cultured When Compared to Native Chromaffin Cells
Source: Front Cell Neurosci. 2017 May 4;11:135. doi: 10.3389/fncel.2017.00135 (PMC5415619; doi:10.3389/fncel.2017.00135)
Supplement: Supplementary file 4 [file Image_4.PDF]

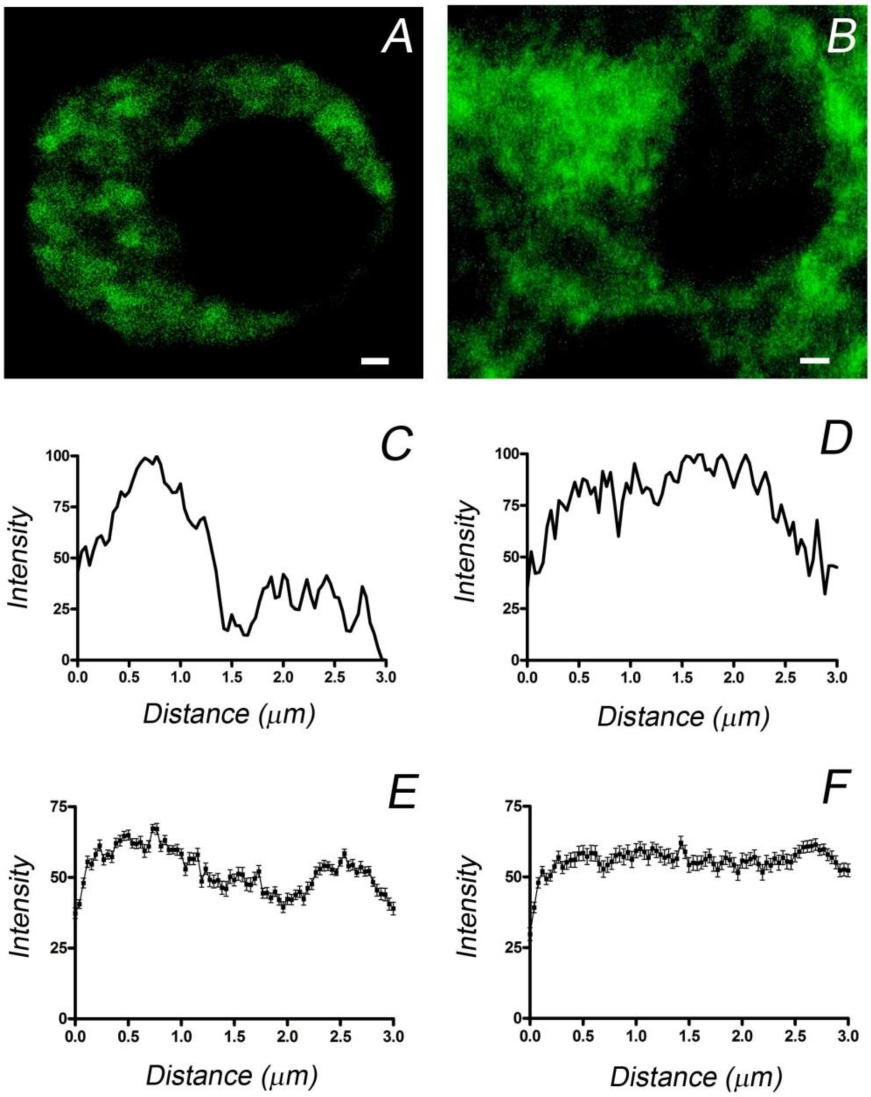

Fig. S 4. Chromaffin granule distribution studied by confocal microscopy in cultured and cells present in the bovine adrenal medulla.

Confocal images of chromaffin granules labelled with anti-dopamine β-hydroxylase representative from experiments performed in cultured chromaffin cells (A) and cells present in the adrenal medulla (B). Example profiles of fluorescence intensity corresponding to the examples depicted for cultured cells (C) and the cells in the adrenal gland (D). The individual fluorescence profiles were averaged to obtain the distribution of granular fluorescence for cultured (n=20 cells) and cells present in the adrenal medulla (F, n=20 cells)). Bars in A and B represent 1 μm.
